# Parent-perceived neighbourhood environment, parenting practices and preschool-aged children physical activity and screen time: a cross-sectional study of two culturally and geographically diverse cities

**DOI:** 10.1186/s12887-022-03377-0

**Published:** 2022-05-27

**Authors:** Ester Cerin, Anthony Barnett, Tom Baranowski, Rebecca E. Lee, Robin R. Mellecker, Yi Nam Suen, Jason A. Mendoza, Deborah I. Thompson, Teresia M. O’Connor

**Affiliations:** 1 Mary MacKillop Institute for Health Research, Australian Catholic University, 215 Spring St, Melbourne, VIC Australia; 2 School of Public Health, The University of Hong Kong, 7 Sassoon Rd., Sandy Bay, Hong Kong, Hong Kong, SAR China; 3 Baker Heart and Diabetes Institute, Melbourne, VIC Australia; 4 Department of Community Medicine, UiT The Artic University of Norway, Tromsø, Norway; 5 USDA/ARS Children’s Nutrition Research Center, Department of Pediatrics, Baylor College of Medicine, 1100 Bates St, Houston, TX 77030 USA; 6 Center for Health Promotion and Disease Prevention, Edson College of Nursing and Health Innovation, Arizona State University, 550 N. 3rd St, Phoenix, AZ85004 USA; 7 Faculty of Education, The University of Hong Kong, Pokfulam Road, Hong Kong, Hong Kong, SAR China; 8 Department of Psychiatry, The University of Hong Kong, Pokfulam Road, Hong Kong, Hong Kong, SAR China; 9 Public Health Sciences Division, Fred Hutchinson Cancer Research Center, 1100 Fairview Ave. N, Seattle, WA USA; 10 Seattle Children’s Research Institute, University of Washington, Seattle, WA USA

**Keywords:** Destinations and facilities, Community cohesion, Informal social control, Parenting practices, Socio-ecological model, Interaction effects, Young children, Sedentary behaviours, Physical activity

## Abstract

**Background:**

Preschool-aged children’s physical activity (PA) and screen time (ST) are important health-related behaviours likely influenced by PA opportunities, parental perceptions of neighbourhood safety and parenting practices pertaining to PA and ST. How these factors interact to impact on young children’s PA and ST, and whether their effects are generalisable across cultures and geographical location is not known. This study addressed these knowledge gaps by conducting pooled analyses of comparable data from two culturally and geographically diverse samples – Chinese parent-child dyads from an ultra-dense city (Hong Kong, China) and Latino parent-child dyads from a low-density city (Houston, USA).

**Methods:**

The analytical sample consisted of 164 Hong Kong Chinese and 84 US Latino parent-child dyads with data on socio-demographic characteristics, parent-perceived neighbourhood destinations and facilities for children’s PA, physical and social safety-related neighbourhood attributes, PA-related parenting practices and child’s ST and accelerometer-assessed PA. Generalised linear models with robust standard errors accounting for neighbourhood-level clustering were used to estimate associations and interaction effects.

**Results:**

Hong Kong Chinese children accumulated less PA than US Latino children, although the latter had more ST. Hong Kong Chinese parents reported more parenting practices promoting inactivity. Neighbourhood PA opportunities were positively related to children’s PA only if parental perceptions of neighbourhood safety were favourable, and the associations of physical neighbourhood environment characteristics with children’s PA and ST depended on PA-related parenting practices. Community cohesion was positively related to children’s PA and negatively related to ST, while parental promotion of ST was positively associated with children’s ST. Correlates of children’s PA and ST did not differ by city.

**Conclusions:**

The substantial differences in activity patterns between Hong Kong Chinese and US Latino preschool-aged children observed in this study are likely due to a combination of cultural and built environmental factors. However, the fact that no between-city differences in correlates of PA and ST were detected indicates that both populations of children are equally affected by parent-perceived neighbourhood environmental characteristics and parenting practices. Overall, this study highlights the importance of considering how various individual-, home- and neighbourhood physical and social factors interact to influence young children’s health-promoting activity levels.

**Supplementary Information:**

The online version contains supplementary material available at 10.1186/s12887-022-03377-0.

## Background

Regular engagement in physical activity (PA) has been shown to improve measures of cardiometabolic health, motor skill development and psychosocial health in preschool-aged children (3–5 years) [1]. In contrast, time spent sedentary, in particular screen time (ST) (e.g., TV viewing), has been associated with unfavourable measures of adiposity, cognitive development and psychosocial health in the same age group [2]. Thus, it is recommended that preschool-aged children engage in at least 180 minutes of a variety of physical activities per day, of which at least 60 minutes should be energetic play, and that their ST be limited to 1 hour per day [3].

The evidence on young children meeting the PA and ST guidelines is mixed and varies across geographical locations and ethnic groups [4–11]. Genuine differences in levels of PA and ST across geographical locations are likely to be primarily due to differences in physical and social environments to which children are exposed [5, 12, 13], including parental attitudes and practices related to PA and ST [4, 14], opportunities for PA and screen-related pursuits [4, 15], and the extent to which parents perceive PA environments to be safe [16, 17]. In this regard, socio-ecological models posit that young children’s PA and sedentary behaviours are shaped by the interaction of individual factors (e.g., child’s sex) and attributes of the home, neighbourhood and preschool physical and social environments [12]. However, whilst a substantial number of studies have examined individual factors, parenting practices and preschool or home characteristics as correlates of young children’s PA [4, 18] and/or ST [19, 20], the role of the neighbourhood physical and social environments remains understudied [4, 18]. This is somewhat at odds with the fact that time spent outdoors is one of the most consistent predictors of PA in this age group [4].

Aspects of the neighbourhood environment that are likely to be most important to young children’s PA are safety and opportunities for PA [21]. The limited evidence on neighbourhood environmental correlates of young children’s PA indicates that young children may benefit from the availability of play/sport equipment and higher levels of traffic safety, whilst the findings on the potential effects of access to various PA places (e.g., garden, parks, playgrounds) and safety from crime have been mixed [18]. Other characteristics of the social neighbourhood environment that may influence parental perceptions of safety, such as neighbourhood informal social control and community cohesion [22, 23], have been examined only in a couple of studies [24, 25]. The evidence on the effects of the neighbourhood environment on young children’s ST is even scarcer than that on PA [26]. However, the fact that more time spent outdoors is one of the best predictors of lower levels of sedentary time in this age group [27] suggests that neighbourhood characteristics that impact outdoor PA may also impact ST in the opposite direction [20, 28].

Research on neighbourhood environmental correlates of PA and ST in preschool-aged children has focused on quantifying main effects [4, 18], overlooking that neighbourhood safety-related attributes may determine the extent to which opportunities for PA in the neighbourhood translate into higher activity levels, as noted in other age groups [29–31] and in a recent qualitative study of parents of preschool-aged children [28]. Whether opportunities for PA in the neighbourhood result in young residents being more physically active may also depend on whether parents encourage or discourage their children to engage in PA and sedentary behaviours [15, 32]. Parenting practices (i.e., specific actions of parents intended to influence their child’s behaviour) related to children’s PA and sedentary behaviours were linked to young children’s PA and ST [4, 14, 26]. They were also found to moderate the associations of environmental factors with PA and sedentary behaviours in school-aged [32, 33] and preschool-aged children [15], although such studies are rare.

To understand how the neighbourhood built and social environments and parenting practices interact to shape young children’s PA and sedentary behaviours, it is valuable to conduct pooled analyses of comparable data from locations with different built environment characteristics and cultures. Multi-country studies can enhance the variability in exposures, allowing for a better characterisation of relationships. They also make it possible to determine the extent to which associations are universal or context specific [34]. However, such studies in young children are lacking, with only a recent one reporting on environmental and parental correlates of preschool-aged children PA and ST in two countries [35].

To address the above-mentioned knowledge gaps, this study used comparable data collected on samples of Chinese and Latino preschool-aged children and their parents living in Hong Kong (China) and Houston (U.S.). Houston and Hong Kong are large metropolises with similar economies and climates but very different levels of density (Houston: 1200 persons/km^2^; Hong Kong: 7140 persons/km^2^) and crime [36], providing large variability in environmental exposures for pooled analyses. Latino and Chinese share important cultural values that foster growth and development in children [37], such as familism, whereby individuals place more importance on the needs of the family as a group than the needs of any individual family member [38, 39]. However, it is not known whether these values translate into them sharing parenting practices that support the development of a healthy active lifestyle in children.

The specific aims of the study were to (1) estimate the contribution of PA- and safety-related aspects of the neighbourhood environment to accelerometer-assessed PA and parent-reported ST in preschool-aged children of different cultural backgrounds (Chinese and Latino) living in an ultra-dense (Hong Kong) and low-density city (Houston); (2) examine the interacting effects of PA- and safety-related aspects of the neighbourhood environment on children’s PA and ST; (3) estimate the added contribution of PA-related parenting practices to children’s PA and ST and their interactions with aspects of the neighbourhood environment; (4) and examine whether the above effects vary by study site and child’s sex. This knowledge is important as it informs the creation of community environments and promotion of parenting practices that support an active and healthy lifestyle in early childhood in different contexts.

## Methods

We used data from two cross-sectional studies conducted in Hong Kong (China) and Houston (U.S.) with comparable measures of socio-demographic characteristics, perceived environmental attributes, PA-related parenting practices and preschool-aged children’s PA and ST. The Hong Kong study recruited a purposive sample of 164 Chinese-speaking parents or primary caregivers (thereafter, parents) and their 3–5 year-old child from maternal health clinics, kindergartens and preschool playgroup centres located in areas cross-stratified by population density and household income [40, 41]. The Houston study recruited a purposive sample of 85 Latino parents and their 3–5 year-old child residing in administrative areas cross-stratified by crime and traffic safety [14]. The stratified sampling strategies aimed at capturing a wide range of neighbourhood environments to more accurately characterise associations of environmental attributes with children’s PA and sedentary behaviours [14, 40–42]. Participants were recruited across all seasons to minimise seasonal effects on activity outcomes. The Hong Kong study was approved by Ethics Committees of the University of Hong Kong and the Department of Health (Hong Kong Special Administrative Region). The Houston study was approved by the Baylor College of Medicine Institutional Review Board. Further details about the recruitment strategies and study designs are provided elsewhere [14, 40, 41].

### Participants and procedures

Hong Kong Chinese parents were approached at selected locations (e.g., maternal health clinics and kindergartens) by research assistants supported by local staff. Hong Kong parents (and their 3–5 year-old child) were eligible to be included in the study unless they were unable to read and write in Chinese or their 3–5 year-old child had a health condition affecting their PA behaviour [41]. Houston Latino parents were recruited through several channels, including community organisations, events, websites and flyers posted at various locations. Houston parents and one of their 3–5 year-old children were eligible to be included in the study if they self-identified as Latino, they were able to read and write in English or Spanish, and their 3–5 year-old child did not have a health condition preventing him/her from participating in PA [14].

All eligible parents provided written informed consent for themselves and for their child to participate. Research staff met the participating parent-child dyad at home (in Hong Kong or Houston) or at the recruitment location (in Hong Kong). Parents self-completed a set of questionnaires in their preferred language (Chinese in Hong Kong; English or Spanish in Houston), for which they received a modest incentive. Parents were provided activity monitors (ActiGraph GT3X or GT3X+ accelerometers) and accessories (activity monitor wear-time log, pouch for the monitor and adjustable elastic belt) for their child, with verbal and written instructions on how to use them. They were instructed that their child should wear the waist-mounted accelerometer above their right hip for seven consecutive days during waking hours, except during water activities and bathing. Parents completed a monitor wear-time log for their child and received an additional modest incentive after providing valid monitor data (as defined below) and wear-time logs. All Hong Kong participants completed the study and provided valid monitor data. In Houston, one participant withdrew from the study and two additional participants did not have valid monitor data after re-wears, leaving 82 participants with valid data [14].

### Measures

The Hong Kong and Houston studies collected parent-reported data on perceived neighbourhood environmental attributes, PA-related parenting practices and socio-demographic characteristics. To allow data pooling, only items that were fully comparable (i.e., same content/meaning) across the two studies were included in the present analyses and self-report measures described below (see Table S1 for examples of items). The internal consistency coefficients of the shorter, harmonised measures and their correlations with the corresponding full-length, original measures employed in the Hong Kong and Houston studies are reported in the Supplementary material (Table S1). The Spanish and Chinese versions of the full-length, original measures have been previously validated in Latino [42] and Hong Kong Chinese parents of preschool-aged children [43].

#### Parent-perceived PA destinations and facilities in the neighbourhood

*Places for Children’s PA* were assessed with a modified 11-item subscale of the Neighbourhood Environment Walkability Scale for Youth (NEWS-Y) [44]. The subscale measures the perceived availability of destinations such as beach, swimming pool, park and playground within a 15-minute walk from home. Scores range from 0 to 11. The test-retest reliability of this measure was excellent in both Latino and Chinese parents [42, 43]. *Availability of Active-Play Equipment* was measured using an 8-item checklist of free or fixed play equipment appropriate for young children with scores ranging from 0 to 8. Test-retest reliability for this measure was 0.62 in Chinese [43] and 0.89 in Latino [42]. As these two measures were originally developed for U.S. English-speaking parents, to achieve cultural equivalence, they were translated into Chinese and Spanish, back-translated into English and reviewed by panels of bilingual experts [42, 43]. Furthermore, the Chinese versions of the measures were examined for cultural and linguistic appropriateness, clarity and comprehensiveness [43] using cognitive interviews seeking to evaluate sources of response errors in self-report measures from the participants’ viewpoint [45]. All items included in this study were deemed culturally appropriate and equivalent to the original measures [42, 43].

#### Parent-perceived physical safety-related attributes of the neighbourhood

*Traffic Hazards* were assessed using five items from the NEWS-Y [44] rated on a 4-point Likert scale. This scale showed substantial repeatability in both populations of parents [42, 43]. *Signs of Physical and Social Disorder* consisted of 16 items rated on a 5-point frequency scale with substantial to excellent test-retest reliability [42, 43]. Scores on each of these measures represent mean ratings on the relevant items. Cultural equivalence of these self-report measures for Latino and Hong Kong Chinese parents of preschool-aged children was ensured using the procedures detailed above [42, 43].

#### Parent-perceived social safety-related attributes of the neighbourhood

We assessed *Community Cohesion* with seven items from the Perceived Neighborhood Scale [46] rated on a 5-point Likert-type scale. This scale gauges feelings of trust, mutual influence and shared social and emotional ties with neighbours. The repeatability of this measure was substantial to excellent in both Chinese [43] and US [47] samples. Cultural equivalence of this measure was achieved through careful translation and cognitive interviews [43]. Aspects of *Neighbourhood Informal Social Control* were measured with seven common items taken from scales specifically developed for the Hong Kong [23, 48] and Houston studies [22]. Four of these items assess *Education and Supervision of Children* and the remaining three represent *Civic Engagement for Neighbourhood Enhancement*. They were rated on a 5-point Likert scale and scores were defined as the mean rating on the items representing each subscale. The full-length version of these subscales showed substantial to excellent repeatability [22, 41]. The assessment of cultural equivalence of the Neighbourhood Informal Social Control items selected for the present study was based on a comparison of the content of the items back-translated in English and their loadings on latent factors from confirmatory factor analyses (CFAs) [22, 23]. We selected items that were identical or nearly identical in content and loaded on comparable latent factors.

#### Parenting practices related to PA and ST

PA-related parenting practices were measured using 19 common items from instruments developed for the two studies [14, 49]. Ten items assessed parenting practices encouraging PA and were deemed to represent *Parental Engagement*. The remaining items measured parenting practices discouraging PA. They were grouped into four subscales: *Restriction for Safety Concerns* (two items); *Psychological Control* (two items); *Promoting Inactivity* (three items); and *Promoting Screen Time* (two items). All items were rated on a 5-point frequency scale. The full-length subscales showed moderate to excellent test-retest reliability in both Latino [14] and Chinese parents [49]. To ensure cultural equivalence of the subscales used in this study, we selected items that were identical or nearly identical in content (based on their back-translated English versions) and measured similar latent factors identified by earlier scale validation work [14, 49].

#### Socio-demographic characteristics

Parents provided information on the child’s characteristics (sex and age) and the amount of time s/he spent outside the home (e.g., in preschool) on an average week. They also provided sociodemographic information about themselves (age, sex, education, employment status, relationship to child, marital status) and the household (number of adults and children, income, highest educational attainment).

#### Child PA and ST

Children’s PA was measured using waist-mounted accelerometers (ActiGraph GT3X/GT3X+) worn on the right hip during waking hours for seven consecutive days. Data were sampled at 15 second epochs [50]. Parents documented accelerometer non-wear time in a log. Log-based non-wear time period and periods of 30 or more minutes of recorded 0 accelerometer counts were removed from analysis. Accelerometer data were considered valid if a child recorded ≥480 min/day of activity data for four or more days, including one weekend day [14]. Participants with an insufficient number of valid days were asked to re-wear the accelerometers for additional days. Pate’s cut-points for preschool-aged children [51] were used to classify accelerometer counts into sedentary (0–37 counts/15 seconds), light-to-vigorous physical activity (≥38 counts/ 15 seconds), representing total PA, and moderate-to-vigorous PA (MVPA) (≥420 counts/ 15 seconds) given that PA guidelines for preschool-aged children recommend the accumulation of three hours per day of any PA, of which at least 60 minutes should be of higher intensity [3]. For the present study, we calculated average daily minutes of total PA, MVPA and sedentary time on weekdays and weekend days.

Child’s ST was measured using a set of five questions (e.g., watching TV, videos, playing videogames) with reference to a typical weekday and weekend day [52]. Parents were asked to report the average time spent on each of the five screen-related behaviours (hours and minutes). For this study, answers to the five questions were summed to create a measure of overall ST on a typical weekday and typical weekend day.

### Data analytic plan

Generalised linear models with robust standard errors accounting for clustering at the administrative unit level were used to estimate the associations. As total PA and ST were approximately normally distributed, Gaussian variance and identity link functions were used to model them, while Gamma variance and logarithmic link functions were used to model positively skewed MVPA. Between-city and between-sex differences in explanatory variables (e.g., parent-perceived neighbourhood attributes) and outcomes (e.g., accelerometer-assessed PA on weekdays) were first assessed. A second set of models estimated the associations of parent-perceived neighbourhood environmental attributes and each of the outcomes. For these models we also estimated whether associations varied by child’s sex and study site (city). We then examined whether the associations of parent-perceived PA destinations and facilities in the neighbourhood with the outcome variables were moderated by safety-related environmental attributes, and probed significant interaction effects by estimating the associations of specific measures of PA destinations/facilities at below-average (1 SD below the mean), average and above-average (1 SD above the mean) values of the moderator (parent-perceived neighbourhood safety-related attribute).

Another set of models estimated the added contribution of PA parenting practices to children’s PA and ST and the moderating effects of parenting practices on the associations between perceived environmental attributes and the outcome variables. Specifically, we examined the moderating effects of Parental Engagement on the associations between PA destination places/facilities and child PA and ST because we hypothesised that parental participation in PA with the child may compensate for the lack of neighbourhood PA opportunities for the child or exert synergistic effects on PA [32]. We also estimated the moderating effects of Restriction for Safety Concerns on the associations between physical safety-related neighbourhood variables and child’s activity measures because safety-related neighbourhood attributes are likely to be more strongly associated with children’s PA and ST if parents limit their children’s PA for safety reasons. Significant interactions were probed by estimating the effects of exposure variables on the outcomes at below-average (1 SD below the mean), average and above-average (1 SD above the mean) values of the moderators. All models were adjusted for socio-demographic covariates, examined for curvilinearity of associations (when appropriate) and heterogeneity of associations across study sites. A probability level of 0.05 was used. No adjustment for multiple significant testing was employed, because the analyses were theory-driven rather than exploratory. In such cases, adjustment for multiple comparisons is not recommended because confirmation by other studies is generally sought for any new discoveries made [53, 54].

## Results

Table 1 reports the socio-demographic characteristics of the two study samples of preschool-aged children and parents. Children were similar in age and sex composition across study sites. While all Hong Kong children attended preschool, nearly a third of Houston children did not. All parents in the Houston study were female, mainly mothers of the participating child, whilst the Hong Kong sample also included fathers (~13%). Hong Kong parents were, on average, older than their Houston counterparts and were more likely to work, be employed full-time and have a tertiary degree. Hong Kong households were also more likely to have a household member with tertiary education. Households in the Hong Kong study tended to have fewer children than households participating in the Houston study (Table 1).

**Table 1:** Sample characteristics by study site

Characteristic	Hong Kong (China) ***n*** = 164	Houston (USA) ***n*** = 82	Between-city differences ***p***-values
***Child’s characteristics***			
Sex, count (%)			.939
Male	91 (55.5%)	45 (54.9%)	
Female	73 (44.5%)	37 (45.1%)	
Age (years), M (SD)	4.3 (0.6)	4.5 (0.6)	.135
Attending preschool, count (%)	164 (100%)	57 (69.5%)	<.001
No. of hours spent outside the home in childcare, day care, preschool, count %			<.001
None	0 (0%)	25 (30.5%)	
Up to 10 hours per week	11 (6.7%)	12 (14.6%)	
11–20 hours per week	59 (36.0%)	12 (14.6%)	
>20 hours per week	94 (57.3%)	33 (40.2%)	
***Caregiver’s characteristics***			
Sex, count (%)			.012
Male	23 (14.0%)	0 (0%)	
Female	141 (86.0%)	82 (100%)	
Age (years), M (SD)	37.6 (5.4)	32.8 (6.7)	<.001
Relationship to child, count (%)			<.001
Mother	138 (84.1%)	79 (96.3%)	
Father	22 (13.4%)	0 (0%)	
Others (female or male relative)	4 (7.8%)	3 (3.7%)	
Education, count (%)			<.001
Less than high school	63 (38.4%)	25 (30.5%)	
Completed high school	32 (19.5%)	41 (50.0%)	
Tertiary education	69 (42.1%)	16 (19.5%)	
Employment status, count (%)			<.001
Not working	73 (44.5%)	51 (62.2%)	
Employed, part-time	14 (8.5%)	14 (17.1%)	
Employed, full-time	44 (26.8%)	15 (18.3%)	
Employed, more than full-time	33 (20.1%)	2 (2.4%)	
***Household characteristics***			
No. of adults (≥19 years), count (%)	2.5 (1.1)	2.2 (0.90)	.151
No. of children (≤18 years), count (%)	1.8 (0.8)	2.9 (1.4)	<.001
Highest education, count (%)			<.001
Less than high school	48 (29.3%)	19 (23.2%)	
Completed high school	34 (20.7%)	47 (57.3%)	
Tertiary education	82 (50.0%)	16 (19.5%)	

Significant between-site differences were found on several measures of perceived neighbourhood environmental attributes and PA-related parenting practices (Table 2 and S2). Parents from Houston reported higher levels of traffic hazards, signs of physical and social disorder, informal social control, child’s PA restriction for safety concerns and promoting screen time. In contrast, parents from Hong Kong reported higher levels of parenting practices promoting inactivity. Large between-site differences were observed in children’s PA and ST, with Hong Kong children accumulating fewer minutes of accelerometer-assessed total PA and MVPA and engaging in less ST than their Houston counterparts. The percentages of children meeting the PA guidelines was much higher in Houston (73–79%) than Hong Kong (10–23%), while the opposite was true for meeting the ST guidelines (Table 2 and S2). In Hong Kong, significant between-sex differences were found in meeting the PA guidelines on weekdays, with girls less likely to do so than boys (Table S2). Small differences were observed in perceived signs of physical and social disorders between parents of boys and girls (Table S2). Girls tended to be slightly less physically active than boys on weekdays (Table S2).

**Table 2:** Descriptive statistics of perceived neighbourhood environmental attributes, parenting practices and children’s physical activity and screen time by city

Variables [range of values]	Hong Kong ***n*** = 168	Houston ***n*** = 82	Between-city differences ***p***-values
***Neighbourhood environmental attributes***			
*PA destinations and facilities*			
Availability of places for children’s PA [0–11]	5.8 (2.4)	5.2 (2.8)	.203
Availability of active-play equipment [0–8]	5.1 (1.9)	5.0 (2.2)	.787
*Physical safety-related attributes*			
Traffic hazards [1–4]	2.4 (0.6)	2.6 (0.7)	.048
Signs of physical and social disorder [1–5]	1.8 (0.6)	2.2 (0.8)	.015
*Social safety-related attributes*			
Community cohesion [1–5]	3.5 (0.6)	3.4 (0.8)	.381
Informal social control – education and supervision of children [1–5]	3.1 (0.6)	3.3 (0.8)	.035
Informal social control – civic engagement for neighbourhood enhancement [1–5]	3.2 (0.6)	3.4 (0.8)	.035
***Parenting practices***			
Parental engagement [1–5]	3.6 (0.6)	3.5 (0.6)	.747
Restrictions for safety concerns [1–5]	1.9 (1.0)	2.7 (1.1)	<.001
Psychological control [1–5]	1.6 (0.8)	1.7 (0.8)	.171
Promoting inactivity [1–5]	1.9 (0.8)	1.7 (0.6)	.046
Promoting screen time [1–5]	2.1 (0.8)	2.3 (0.8)	.018
***Child’s physical activity, sedentary time and screen time***			
*Average weekday*			
Total PA (min/day) [accelerometer-assessed]	128.3 (38.0)	334.6 (59.0)	<.001
MVPA (min/day) [accelerometer-assessed]	53.9 (19.5)	81.5 (36.7)	<.001
Meeting physical activity guidelines, %	10.4	79.3	<.001
Sedentary time (min/day) [accelerometer-assessed]	563.4 (77.0)	388.0 (82.3)	<.001
Accelerometer wear time (hrs/day)	11.6 (1.4)	12.0 (1.3)	.010
Screen time (hrs/day) [parent-reported]	2.8 (2.2)	5.0 (2.8)	<.001
Meeting screen time guidelines, %	19.5	2.4	.003
*Average weekend day*			
Total PA (min/day) [accelerometer-assessed]	151.8 (52.6)	318.1 (85.7)	<.001
MVPA (min/day) [accelerometer-assessed]	69.6 (30.8)	87.1 (48.8)	.003
Meeting physical activity guidelines %	22.6	73.2	<.001
Sedentary time (min/day) [accelerometer-assessed]	531.0 (96.1)	323.8 (83.6)	<.001
Accelerometer wear time (min/day)	11.4 (1.7)	10.7 (1.9)	.009
Screen time (hours/day) [parent-reported]	2.9 (2.1)	4.2 (2.6)	.022
Meeting screen time guidelines	21.3	11.0	.035

Numbers represent means and standard deviations (in brackets) unless otherwise stated

*PA* physical activity, *MVPA* moderate-to-vigorous physical activity, *ST* screen time. Meeting physical activity guidelines means accumulating 180 min of total PA per day of which 60 minutes are MVPA. Meeting screen time guidelines means accumulating up to 1 hour of screen time per day. *p*-values were derived from generalised linear models with city and sex (and, when appropriate, their interaction and accelerometer wear time) as predictors of the variables listed in the table. Table S2 also reports the significant effects of sex and sex by city

### Perceived environmental attributes as correlates of PA and ST

No significant associations of perceived physical aspects of the neighbourhood environment with PA on weekdays were observed (Table 3). However, availability of places for PA was positively related to total PA on weekend days (Table 3). Three significant interactions between PA destinations/facilities and safety-related neighbourhood attributes were observed (Table 4). The association of availability of places for PA with total PA on weekends and MVPA on weekdays was positive and stronger in children with parents reporting higher levels of civic engagement for neighbourhood enhancement. Availability of active play-equipment was positively associated with total PA on weekend days but only in children whose parents reported below-average levels of traffic hazards in the neighbourhood.

**Table 3:** Associations of caregivers’ perceptions of neighbourhood environmental attributes and physical-activity-related parenting practices with preschool-aged children’s physical activity and screen time: main and child sex-specific effects

Correlate	Measurement period	Total PA (min/day) [accelerometer-assessed]	MVPA (min/day) [accelerometer-assessed]	Screen time (hr/day) [parent-reported]
		*b* (95% CI)	e^*b*^ (95% CI)	*b* (95% CI)
***Neighbourhood environmental attribute***				
*PA destinations and facilities*				
Availability of places for children’s PA	Weekday	0.79 (−1.40, 2.98)	1.01 (0.99, 1.03)	0.04 (−0.08, 0.16)
	Weekend	3.16 (0.73, 5.59)^*^	1.01 (0.99, 1.03)	−0.04 (−0.15, 0.07)
Availability of active-play equipment in the home/neighbourhood	Weekday	−0.24 (−2.98, 2.51)	1.00 (0.98, 1.02)	0.17 (−0.02, 0.36)
	Weekend	1.41 (−1.93, 4.75)	1.00 (0.98, 1.03)	0.06 (−0.13, 0.24)
*Physical safety-related attributes*				
Traffic hazards	Weekday	0.83 (−9.27, 10.92)	0.99 (0.91, 1.07)	0.06 (−0.50, 0.50)
	Weekend	−0.74 (−13.30, 11.82)	0.98 (0.89, 1.09)	0.15 (−0.50, 0.80)
Signs of physical and social disorder	Weekday	0.45 (−10.50, 11.40)	1.01 (0.92, 1.11)	0.02 (−0.45, 0.50)
	Weekend	4.31 (−8.52, 17.13)	1.01 (0.91, 1.11)	0.23 (−0.30, 0.75)
*Social safety-related attributes*				
Community cohesion	Weekday	10.17 (1.80, 18.54)^*^	1.04 (0.96, 1.13)	−0.44 (−0.85, −0.02)^*^
	Weekend	13.51 (3.25, 25.58)^**^	1.09 (1.02, 1.17)^**^	0.02 (−0.48, 0.51)
Informal social control – education and supervision of children	Weekday	−6.89 (−17.62, 3.83)	0.93 (0.85, 1.01)	0.32 (−0.20, 0.83)
	Weekend	M: −2.20 (−17.84, 13.44) F: −29.66 (−49.43, −9.89)^**^	0.93 (0.84, 1.02)	−0.05 (−0.52, 0.42)
Informal social control – civic engagement for neighbourhood enhancement	Weekday	5.51 (−4.94, 15.95)	1.02 (0.94, 1.11)	−0.17 (−0.71, 0.37)
	Weekend	M: −3.65 (−15.96, 8.66) F: 23.38 (3.47, 43.30)^**^	1.09 (1.01, 1.20)^*^	0.14 (−0.31, 0.59)
***PA-related parenting practices***				
Parental engagement	Weekday	−4.63 (13.90, 4.62)	0.99 (0.91, 1.08)	−0.43 (−0.83, −0.03)^*^
	Weekend	0.23 (−1.76, 11.22)	1.07 (0.98, 1.18)	0.14 (−0.32, 0.60)
Restrictions for safety concerns	Weekday	0.41 (−5.02, 5.84)	0.99 (0.94, 1.03)	0.09 (−0.18, 0.36)
	Weekend	0.79 (−6.26, 7.83)	1.01 (0.96, 1.07)	0.22 (−0.13, 0.56)
Psychological control	Weekday	M: 10.05 (0.42, 19.68)^*^ F: −5.72 (−13.50, 2.06)	M: 1.10 (1.01, 1.20)^*^ F: 0.95 (0.90, 1.02)	−0.28 (−0.68, 0.13)
	Weekend	−0.67 (−11.42, 10.08)	0.90 (0.91, 1.07)	−0.03 (−0.44, 0.38)
Promoting inactivity	Weekday	2.59 (−6.12, 11.31)	1.04 (0.96, 1.12)	0.19 (−0.27, 0.65)
	Weekend	−3.23 (−14.81, 8.34)	0.95 (0.86, 1.04)	0.20 (−0.34, 0.73)
Promoting screen time	Weekday	−5.68 (−13.14, 1.79)	0.97 (0.94, 1.03)	0.99 (0.69, 1.29)^***^
	Weekend	−6.89 (−16.50, 2.72)	0.95 (0.89, 1.02)	0.59 (0.27, 0.90)^***^

*M* males, *F* females, *b* estimate of unstandardised regression coefficient, e^*b*^, exponentiated estimate of unstandardised regression coefficient, *CI* confidence interval, *PA* physical activity, *MVPA* moderate-to-vigorous physical activity

^*^p < .05; ^**^p < .01; ^***^p < .001. All models were adjusted for socio-demographic characteristics. Models of total PA and MVPA were adjusted for average accelerometer wear time. Regression coefficients of neighbourhood environmental attributes were not adjusted for PA-related parenting practices. However, regression coefficients of PA-related parenting practices were adjusted for neighbourhood environmental attributes

**Table 4:** Interaction effects between parent-perceived neighbourhood physical activity places and facilities, safety-related attributes and physical-activity-related parenting practices on preschool-aged children’s physical activity and screen time

Neighbourhood environmental attribute	Moderator	Outcome	Association at below average value of moderator	Association at average value of moderator	Association at above average value of moderator
			Reg. coef. (95% CI)	Reg. coef. (95% CI)	Reg. coef. (95% CI)
Availability of places for children’s PA	Informal social control - civic engagement for neighbourhood enhancement	MVPA – weekday	e^*b*^ = 0.99 (0.96, 1.01)	e^*b*^ = 1.01 (0.99, 1.02)	**e**^***b***^ = 1.03^*^(1.01, 1.05)
Availability of places for children’s PA	Informal social control - civic engagement for neighbourhood enhancement	Total PA – weekend day	*b* = 0.24 (−2.84, 3.33)	***b*** = 2.91^*^(0.47, 5.35)	***b*** = 5.58^**^(1.86, 9.31)
Availability of active-play equipment	Traffic hazards	Total PA – weekend day	***b*** = 6.36^*^(0.37, 12.34)	*b* = 2.02 (−1.46, 5.51)	*b* = −2.31 (−6.34, 1.72)
Availability of places for children’s PA	Parental engagement	Total PA - weekday	***b*** = 3.32^*^(0.69, 5.94)	*b* = 1.02 (−1.14, 3.18)	*b* = −1.28 (−4.22, 1.66)
Availability of active-play equipment	Parental engagement	MVPA - weekday	**e**^***b***^ = 0.96^*^(0.94, 0.99)	e^*b*^ = 1.00 (0.98, 1.02)	**e**^***b***^ = 1.05^*^(1.00, 1.11)
Signs of physical and social disorder	Restrictions for safety concerns	Screen time – weekend day	*b* = −0.46 (−1.19, 0.27)	*b* = 0.07 (−0.38, 0.53)	***b*** = 0.61^*^(0.11, 1.10)

*b*, estimate of unstandardised regression coefficient; e^*b*^, exponentiated estimate of unstandardised regression coefficient; *CI* confidence interval, *PA* physical activity, *MVPA* moderate-to-vigorous physical activity

^*^p < .05; ^**^ *p* < .01. All models were adjusted for socio-demographic characteristics. Models of total PA and MVPA were adjusted for average accelerometer wear time. Regression coefficients of neighbourhood environmental attributes moderated by other environmental attributes were not adjusted for PA-related parenting practices. Below and above average value of moderator means respectively 1 standard deviation below and above the moderator’s mean. Only statistically significant (*p* < .05) interaction effects are reported in this table. Table S3 reports all tested interaction effects (statistically significant and not significant)

A greater number of significant associations were observed between perceived neighbourhood social safety-related attributes and children’s PA and ST (Table 3). Community cohesion was positively related to accelerometer-assessed PA and MVPA. A weak negative association between community cohesion and screen time on weekdays was also observed. The associations of aspects of informal social control with children’s activity measures tended to depend on child’s sex. Education and supervision of children was negatively related to total PA only in girls. Similar sex-specific findings were observed for the other aspect of informal social control – civic engagement for neighbourhood enhancement – but in the opposite direction. Associations were generally stronger for weekend measures of activity. No significant between-site differences were observed in any of the above associations.

### Parenting practices and children’s PA and ST

After adjustment for perceived environmental attributes, promoting ST was positively related to parent-reported child’s ST (Table 3). Higher psychological control was associated with higher levels of activity but only in boys and on weekdays. While parental engagement in PA was negatively associated with weekday ST only (Table 3), it also moderated the association of availability of places for PA with child’s total PA on weekdays and of availability of active-play equipment with MVPA on weekdays (Table 4). Specifically, availability of places for PA was positively related to total PA only in those with parents reporting below-average levels of parental engagement. The opposite was observed with respect to availability of active-play equipment and MVPA on weekdays, where a positive association was observed in children of parents with higher levels of parental engagement. Promoting inactivity and restrictions for safety concerns were unrelated to children’s PA and ST. However, restrictions for safety concerns moderated the associations of signs of physical and social disorder with weekend ST, where positive associations were observed only at above-average levels of restrictions (Table 4).

## Discussion

This study examined the associations of parent-perceived PA- and safety-related aspects of the neighbourhood environment and PA-related parenting practices with PA and ST in Chinese and Latino preschool-aged children living in an ultra-dense (Hong Kong, China) and low-density city (Houston, USA). By pooling and analysing comparable data from two cities with very diverse built environments and cultures, we were able to examine the extent to which physical and social environmental correlates of preschool-aged children’s PA and ST may be generalisable across geographical locations and cultures. While we did not find sufficient evidence that associations varied by city, we observed several between-city differences in safety-related environmental attributes, PA-related parenting practices and children’s PA and ST. Hong Kong Chinese parents reported higher levels of neighbourhood traffic safety and safety from crime than did their Latino counterparts in Houston, and, perhaps consequently, lower levels of parenting practices restricting children’s PA due to safety concerns. In line with these findings, Hong Kong and Houston were respectively categorised as ‘very low’ and ‘high’ on a global crime index [55, 56], and Houston had a traffic index value 44% higher than Hong Kong [57, 58]. Thus, it is likely that the observed differences in parental perceptions of neighbourhood safety and associated parenting practices were at least, in part, due to objective differences in levels of traffic safety and safety from crime between the two cities.

Hong Kong parents reported using parenting practices promoting inactivity more frequently, and parenting practices promoting ST less frequently, than the US Latino parents. Between-city differences in children’s PA and ST mirrored these findings, with Latino children accumulating, for example, 168% more PA and 79% more ST on weekdays than Hong Kong children. It has been reported that Hong Kong preschool-aged children engage in low levels of PA by other recent studies [7, 59]. For example, Feng and colleagues found that only 14.5% of Hong Kong preschool-aged children met the PA guidelines. The low levels of PA in Hong Kong young children have been attributed to three main reasons: (1) kindergartens having small play areas [60]; (2) parents’ not having the time to play with their children due to work commitments [16]; and (3) Hong Kong parents being influenced by Confucianism, the cornerstone of traditional Chinese culture, which prioritises academic activities over play and PA [61]. It also should be noted that Hong Kong families live in very small high-rise apartments that restrict children’s opportunities to engage in active play. Data from the Hong Kong arm of this study identified apartment size as one of the strongest predictors of accelerometer-assessed PA and sedentary time in Hong Kong preschool-aged children, with those living in large apartments (e.g., 1200 sqf.) accumulating 27% more MVPA than those living in the typical apartment (400 sqf.) [40]. With regard to the high levels of PA observed in US Latino children in this study, we note that even higher levels of PA have been reported in other US samples of preschool-aged children [62] and samples from countries similar to the US in urban form, population density and culture (Australia) [63].

The fact that US Latino children had higher levels of ST than their Hong Kong counterparts is not surprising. Previous studies have reported higher ST in US [62] than Hong Kong young children [7]. Eastern Asian parents typically exert more control and supervision over their children than Western parents [64]. This applies to ST, as shown in another study of Hong Kong preschool-aged children [65].

### The neighbourhood physical environment and children’s PA and ST

We found that perceived availability of active-play equipment and places for PA were both positively related to total PA and MVPA but only in subgroups of children with parents reporting specific levels of neighbourhood safety-related features and parental engagement in PA. These findings suggest that neighbourhoods with age-appropriate opportunities for PA do not necessarily result in young children being more physically active. To have an impact, places for PA need to be embedded in communities collectively committed to creating a safe environment for young children. The fact that previous studies reported mixed findings about the importance of neighbourhood places for PA for young children’s activity levels [18] may be due to studies not considering aspects of neighbourhood safety as potential moderators. This remains a topic requiring further investigation.

Associations between neighbourhood opportunities for PA and children’s PA also depended on how much parents encouraged their children to be physically active through participatory engagement. Positive associations between availability of places for PA and total PA on weekdays were detected only in children of parents with low levels of participatory engagement, who may have generally preferred taking their young children to age-appropriate places for PA in the community and being passive observers. In contrast, parents willing to engage in PA with their young children on weekdays (possibly after working hours) might have done that primarily at home. This would explain why availability of places for PA in the neighbourhood was unrelated to PA in children of parents reporting higher levels of participatory engagement in PA. Future studies could investigate how participatory engagement may influence family PA in the home and neighbourhood.

We observed a synergistic moderating effect of parental engagement in child’s PA on the association between availability of active-play equipment and child’s MVPA on weekdays. Availability of active-play equipment was positively associated with MVPA only at high levels of parental engagement, and negatively associated with MVPA at low levels of parental engagement. Although children may participate in unstructured play in the local playground or park without relying on parental engagement, they may need the assistance of an adult if they wish to play with various pieces of equipment (e.g., ball, bicycle). Thus, having access to a variety of active-play equipment is more likely to result in young children being more active if their parents engage with them in the activity. In contrast, children of parents with low levels of participatory engagement may be negatively affected by a wide range of active-play equipment. In fact, there is evidence that young children play for longer periods of time if they have access to a limited rather than large number of toys [66].

In contrast to previous studies [18], perceived neighbourhood traffic safety did not emerge as an important correlate of children’s PA; although, as mentioned earlier, it moderated the association with availability of active-play equipment in the expected direction. These inconsistent findings could be due to methodological, contextual and/or cultural differences between studies [67–69], such as the availability of safe places for PA in the neighbourhood. For example, in this study, over 75% of parents reported having easy access to more than three types of places for children’s PA (indoor recreation or exercise facilities, friend/relative’s house and playgrounds) that are typically traffic-safe, making it less likely for parental traffic safety concerns to impact on children’s PA.

We found that parental perceptions of neighbourhood disorder were unrelated to children’s PA. However, they were positively associated with ST on weekend days in children of parents who restricted their children’s PA for safety concerns. Parents who consider the neighbourhood a dangerous place where children could get hurt are more likely to keep their young children at home watching TV or playing videogames. Other studies have reported higher levels of ST in preschool-aged children of parents with unfavourable perceptions of neighbourhood safety [19, 24, 70]. However, these studies did not consider PA-related parenting practices. To create an environment that supports a healthy and active lifestyle in young children it is important to understand how parents’ actions and perceptions of the environment interact to guide children’s behaviour [32].

### The neighbourhood and home social environments and children’s PA and ST

Social safety-related neighbourhood attributes, especially community cohesion, emerged as stronger correlates of children’s PA and ST than their physical counterparts. Two other studies of preschool-aged children reported similar findings, with social capital measures like those used in this study being positively associated with outdoor play and meeting the PA guidelines [24, 25]. One of these studies also reported negative associations with ST [24]. Community cohesion and neighbourhood informal social control are the foundation of a safe and strong community [46]. Community cohesion is typified by strong social networks, a sense of belonging, and mutual trust and solidarity among neighbours. These neighbourhood social characteristics may help young children be more physically active and spend less time in front of screens by providing more social and active-play opportunities with neighbouring children and adults, and by reducing parental safety concerns related to traffic and crime.

It is interesting that the two aspects of neighbourhood informal social control examined in this study showed different relationships with children’s PA, with civic engagement for the creation of a safe environment being more influential and beneficial than informal social control defined as education and supervision of neighbourhood children. While community activism focused on enhancing the neighbourhood environment for young children implies a positive attitude towards children’s playing outdoors, this cannot be said for children’s education and supervision. In fact, we found a negative relationship between the latter aspect of informal social control and girls’ total PA on weekend days. Higher levels of neighbourhood informal education and supervision of local children have been found to correlate with higher levels of perceived safety from crime and traffic [22, 23]. Thus, the negative relationship between informal supervision of neighbourhood children and PA in young girls cannot be attributed to lower levels of neighbourhood safety. This specific dimension of informal social control may be an indicator of neighbourhood social norms in favour of overprotective behaviours towards children that restrict their engagement in outdoor active play [71].

Apart from examining neighbourhood social environmental correlates of young children’s PA and ST, we investigated the added contribution of home-level social factors – namely, PA-related parenting practices. While parental promotion of ST was positively associated with children’s ST on both weekdays and weekend days, other parenting practices showed sporadic, weak associations with children’s PA and ST, including a negative association between parental engagement in PA and ST on weekdays. It should be noted that parenting practices and children’s ST were both parent-reported, while children’s total PA and MVPA were measured using accelerometers. Thus, the stronger associations of parental promotion of ST and engagement in children’s PA with children’s ST may have been in part due to common method bias [72]. However, subsequent auxiliary analyses revealed positive relationships between parental promotion of ST and children’s accelerometer-assessed sedentary time on weekdays (*b* = 10.30; 95% CI: 0.11, 20.48; *p* = .048) and weekend days (*b* = 10.21; 95% CI: 0.91, 19.51; *p* = .031), supporting the presence of substantive effects. The current evidence about the impact of various parenting practices on preschool-aged children’s PA and ST is mixed [4, 19], with several authors acknowledging that parenting practices need to be studied in conjunction with other child-, home- and neighbourhood-level interacting contextual factors [15, 28, 32]. The findings from this study support such interactional approach given that few main effects of parenting practices on children’s PA and ST emerged.

### Study strengths and weaknesses

Beyond addressing substantive gaps in the literature on environmental correlates of preschool-aged children’s PA and ST, this study has several other strengths. First, by pooling comparable data from samples located in two geographically and culturally diverse cities (Hong Kong, China; Houston, US), we expanded the variability of exposures and outcomes and were able to test the generalisability of associations across contexts. Second, we used measures of the neighbourhood environment and PA-related parenting practices that were develop for and validated in the target populations. Third, the two-stage stratified sampling strategy employed in this study maximised the variability in environmental exposures within each study site and helped achieve a relatively balanced representation of socio-economic strata. Study limitations include the relatively small sample sizes in the study locations limiting the ability to estimate between-site differences in associations; the non-probabilistic sampling; the under-representation of fathers in the samples; the inability to assess measurement equivalence across the two samples using robust statistical methods, such as Item Response Theory; the cross-sectional nature of the study precluding casual inference; the lack of comparable objective environmental data on the neighbourhood environment; the employment of several parent-reported measures prone to reporting biases, including socially desirable responses.

### Practical implications

Several practical implications can be drawn from the present study. To increase young children’s PA in Hong Kong, public health programs should improve parents’ attitudes towards children’s PA and provide information on safe places where children can engage in active play. In contrast, parents of Latino young children in Houston may benefit more from understanding the health benefits of reducing children’s ST and learning effective strategies to do so (e.g., by not actively promoting ST). This study also suggests that, irrespective of culture and geographical context, to increase preschool-aged children’s PA and reduce their ST, it is important to create safe and cohesive communities with good access to places for PA and active-play equipment where parents can engage in active play with their children.

## Conclusions

This study highlights the importance of considering how various individual-, home- and neighbourhood physical and social factors interact to influence young children’s health-promoting activity levels across various cultures and geographical locations. Neighbourhood opportunities for PA were positively related to children’s PA only if parental perceptions of neighbourhood safety were favourable, and the associations of physical aspects of the neighbourhood environment with children’s PA and ST depended on PA-related parenting practices. The only perceived environmental factor that showed consistent positive main effects on children’s activity levels was community cohesion, while parental promotion of ST was consistently associated with children engaging in more ST. Although marked between-city differences were observed in children’s activity patterns, likely due to cultural factors (promotion of inactivity) and housing conditions (lack of indoor space for PA), we did not find sufficient evidence of between-city differences in correlates of children’s PA and ST. Overall, these findings indicate that, to develop a healthy, active lifestyle, young children need PA-friendly and cohesive community environments as well as parental support for PA.

Given the general dearth of findings on the moderating role of neighbourhood safety and PA-related parenting practices in the effects of access to places for PA with preschool-aged children’s PA and ST, more studies with larger representative samples addressing these issues are needed. Ideally, these studies should be longitudinal, include both perceived and objective measures of neighbourhood attributes, cover various cultures and geographical regions, and use comparable protocols so that pooled analyses can be performed. Future studies should also have a more balanced representation of fathers in the sample and investigate the extent to which parental participatory engagement in children’s active play influences children’s PA in different contexts, i.e., in the home and neighbourhood.

## Supplementary Information

**Additional file 1: Table S1**. List of measures and their internal consistency.

**Additional file 2: Table S2**. Descriptive statistics of perceived neighbourhood environmental attributes, parenting practices and children’s physical activity and screen time by city and child’s sex.

**Additional file 3: Table S3**. Interaction effects between parent-perceived neighbourhood physical activity places and facilities, safety-related attributes and physical-activity-related parenting practices on preschool-aged children’s physical activity and screen time.

## Data Availability

The datasets analysed during the current study are not publicly available due to lack of participant consent to share data outside the team of investigators but are available from the corresponding author on reasonable request.

## Abbreviations

CFAs: Confirmatory factor analyses
CI: Confidence intervals
MVPA: Moderate-to-vigorous physical activity
NEWS-Y: Neighborhood Environment Walkability Scale - Youth
PA: Physical activity
SD: Standard deviation
ST: Screen time
US: United States
