# Supplementary material for: Parent-perceived neighbourhood environment, parenting practices and preschool-aged children physical activity and screen time: a cross-sectional study of two culturally and geographically diverse cities
Source: BMC Pediatr. 2022 May 27;22:309. doi: 10.1186/s12887-022-03377-0 (PMC9137173; doi:10.1186/s12887-022-03377-0)
Supplement: Supplementary file 1 — Additional file 1: Table S1. List of measures and their internal consistency. [file 12887_2022_3377_MOESM1_ESM.docx]

| **Measures** (source) [# of items] | **“*Instructions to respondents*” and response scale**  **[example of items]** | **Cronbach’s alpha (correlation with original (sub)scale)** | | |
| --- | --- | --- | --- | --- |
| **Physical activity (PA)-related parenting practices** | | Hong Kong | | Houston |
| Physical Activity Parental Practices for Preschoolers **(**O’Connor, Cerin, Hughes, Robles, Thompson, Mendoza, Baranowski, & Lee, 2014; Suen, Cerin, Barnett, Huang, & Mellecker, 2017) | “*How often do you …*”  5-point frequency scale (1 = rarely; 5 = frequently/always) |  | | |
| *Practices that encourage child’s PA subscales* |  |  | | |
| Parental engagement [10] | [… dance with your child?] | 0.80  (0.98) | 0.84  (0.98) | |
| *Practices that discourage child’s PA subscales* |  |  | | |
| Restrictions for safety concerns [2] | [… not let your child outside because you are worried about traffic?] | 0.78  (0.84) | 0.67  (0.94) | |
| Psychological control [2] | [… tell your child he/she will get hurt if he/she plays actively?] | 0.60  (0.69) | 0.67  (0.75) | |
| Promoting inactivity* [3] | [… drive your child when walking was an easy option?] | 0.63  (1.00) | 0.50  (1.00) | |
| Promoting screen time [2] | [… allow your child to watch TV for long periods of time?] | 0.71  (1.00) | 0.64  (0.94) | |
| **Perceived neighbourhood environmental factors** |  |  | | |
| PA-related informal social control (Cerin, O’Connor, Mendoza, Thompson, Lee, Hughes, & Baranowski, 2015; Cerin, Suen, Barnett, Huang, & Mellecker, 2017) | “*Choose the best answer that describes people in your neighbourhood. How much do you agree or disagree with the following statements?*”  5-point Likert scale (1 = strongly disagree; 5 = strongly agree) |  |  |  |
|  |  |  | | |
| *Civil engagement for neighbourhood enhancement [3]* | [People in my neighbourhood work with the city to reduce speed limits in our neighbourhood] | 0.75  (0.92) | 0.74  (0.92) | |
| *Education and supervision of children [4]* | [People in my neighbourhood supervise the neighbourhood children at all times] | 0.70  (0.70) | 0.82  (0.92) | |
| Neighbourhood community cohesion* (Martinez, Black, & Starr, 2002; Suen, Cerin, Huang, & Mellecker, 2015) [7] | “*How much do you agree or disagree with the following statements?*”  5-point Likert scale (1 = strongly disagree; 5 = strongly agree)  [People trust each other in my neighbourhood] | 0.72 | 0.89 | |
| Signs of physical and social disorder (O’Connor, Cerin, Lee, Parker, Chen, Hughes, Mendoza, & Baranowski, 2014; Suen, Cerin, Huang, & Mellecker, 2015) [16] | “*How often do you see the following in your neighbourhood?*”  5-point frequency scale (1 = never; 5 = frequently)  [Abandoned cars] | 0.88  (0.99) | 0.93  (1.00) | |
| Traffic hazards (O’Connor, Cerin, Lee, Parker, Chen, Hughes, Mendoza, & Baranowski, 2014; Suen, Cerin, Huang, & Mellecker, 2015) [5] | “*How much do you agree or disagree with the following statements?*”  4-point Likert scale (1 = strongly disagree; 5 = strongly agree)  [Most drivers go faster than the posted speed limits] | 0.68  (0.97) | 0.70  (0.96) | |
| Availability of active-play equipment* (O’Connor, Cerin, Lee, Parker, Chen, Hughes, Mendoza, & Baranowski, 2014; Suen, Cerin, Huang, & Mellecker, 2015) [8] | “*Please indicate whether the following is available to your 3-5 year old child*”  Response: At home (1 point); In the neighbourhood (1 point); Outside the neighbourhood (0 point)  [Roller skates, skateboard, scooter] | NA | | |
| Availability of places for children’s PA* (O’Connor, Cerin, Lee, Parker, Chen, Hughes, Mendoza, & Baranowski, 2014; Suen, Cerin, Huang, & Mellecker, 2015) [11] | “*Please indicate whether the following is available to your 3-5 year old child in your neighbourhood*”  Response: Yes; No  [Large public park] | NA | | |

*Note.* PA = physical activity; NA = not applicable (because checklist/index); * same as original (sub)scale
