# Supplementary material for: Parent-perceived neighbourhood environment, parenting practices and preschool-aged children physical activity and screen time: a cross-sectional study of two culturally and geographically diverse cities
Source: BMC Pediatr. 2022 May 27;22:309. doi: 10.1186/s12887-022-03377-0 (PMC9137173; doi:10.1186/s12887-022-03377-0)
Supplement: Supplementary file 2 — Additional file 2: Table S2. Descriptive statistics of perceived neighbourhood environmental attributes, parenting practices and children’s physical activity and screen time by city and child’s sex. [file 12887_2022_3377_MOESM2_ESM.docx]

|  | Hong Kong | | Houston | | Differences  between … |
| --- | --- | --- | --- | --- | --- |
| **Variables [range of values]** | Male | Female | Male | Female |  |
|  | n = 91 | n = 73 | n = 45 | n = 37 |  |
| ***Neighbourhood environmental attributes*** |  |  |  |  |  |
| *PA destinations and facilities* |  |  |  |  |  |
| Availability of places for children’s PA [0-11] | 5.9 (2.5) | 5.7 (2.4) | 5.2 (2.9) | 5.2 (2.7) | - |
| Availability of active-play equipment [0-8] | 5.1 (1.9) | 5.0 (1.8) | 4.8 (2.3) | 5.2 (2.2) | - |
| *Physical safety-related attributes* |  |  |  |  |  |
| Traffic hazards [1-4] | 2.4 (0.5) | 2.3 (0.6) | 2.7 (0.7) | 2.6 (0.6) | Cities: *p*=.048 |
| Signs of physical and social disorder [1-5] | 1.9 (0.7) | 1.7 (0.5) | 2.3 (0.9) | 2.0 (0.8) | Cities: *p*=.015  Sexes: *p*=.048 |
| *Social safety-related attributes* |  |  |  |  |  |
| Community cohesion [1-5] | 3.5 (0.5) | 3.5 (0.6) | 3.4 (0.9) | 3.4 (0.7) | - |
| Informal social control – education and supervision of children [1-5] | 3.1 (0.6) | 3.2 (0.7) | 3.4 (0.8) | 3.2 (0.9) | Cities: *p*=.035 |
| Informal social control – civic engagement for neighbourhood enhancement [1-5] | 3.2 (0.6) | 3.2 (0.7) | 3.5 (0.7) | 3.4 (0.9) | Cities: *p*=.035 |
| ***Parenting practices*** |  |  |  |  |  |
| Parental engagement [1-5] | 3.5 (0.6) | 3.6 (0.6) | 3.6 (0.6) | 3.4 (0.6) | - |
| Restrictions for safety concerns [1-5] | 2.0 (1.0) | 1.8 (0.9) | 2.8 (1.1) | 2.5 (1.0) | Cities: *p*<.001 |
| Psychological control [1-5] | 1.6 (0.7) | 1.6 (0.8) | 1.7 (0.8) | 1.8 (0.8) | - |
| Promoting inactivity [1-5] | 2.0 (0.8) | 1.8 (0.7) | 1.7 (0.6) | 1.8 (0.6) | Cities: *p*=.046 |
| Promoting screen time [1-5] | 2.1 (0.8) | 2.0 (0.7) | 2.3 (0.8) | 2.3 (0.9) | Cities: *p*=.018 |
| ***Child’s physical activity and screen time*** |  |  |  |  |  |
| *Average weekday* |  |  |  |  |  |
| Total PA (min/day) [accelerometer-assessed] | 134.9 (40.9) | 120.0 (32.3) | 331.6 (59.4) | 338.3 (59.1) | Cities: *p*<.001  Sexes: *p*=.030 |
| MVPA (min/day) [accelerometer-assessed] | 56.6 (21.6) | 50.5 (16.2) | 79.4 (24.7) | 84.2 (47.6) | Cities: *p*<.001 |
| Meeting physical activity guidelines, % | 15.4 | 4.1 | 77.8 | 81.1 | Cities: *p*<.001  City by sex: *p*=.032 |
| Accelerometer wear time (hrs/day) | 11.7 (1.3) | 11.4 (1.5) | 11.9 (1.4) | 12.2 (1.1) | - |
| Screen time (hrs/day) [parent-reported] | 2.7 (2.3) | 2.8 (2.0) | 5.0 (3.0) | 5.0 (2.5) | Cities: *p*<.001 |
| Meeting screen time guidelines, % | 23.1 | 15.1 | 2.2 | 2.7 | Cities: *p*=.003 |
| *Average weekend day* |  |  |  |  |  |
| Total PA (min/day) [accelerometer-assessed] | 156.5 (57.1) | 145.8 (46.2) | 336.0 (77.4) | 296.3 (91.1) | Cities: *p*<.001 |
| MVPA (min/day) [accelerometer-assessed] | 71.7 (33.0) | 66.9 (27.9) | 91.8 (35.4) | 81.3 (61.3) | Cities: *p*=.003 |
| Meeting physical activity guidelines % | 24.2 | 20.6 | 82.2 | 62.2 | Cities: *p*<.001 |
| Accelerometer wear time (min/day) | 11.4 (1.7) | 11.3 (1.7) | 11.0 (1.7) | 10.3 (2.1) | - |
| Screen time (hours/day) [parent-reported] | 3.0 (2.2) | 2.9 (2.0) | 4.0 (2.6) | 4.5 (2.5) | Cities: *p*=.022 |
| Meeting screen time guidelines | 20.9 | 21.9 | 8.8 | 13.5 | Cities: *p*=.035 |

*Notes.* Numbers represent means and standard deviations (in brackets) unless otherwise stated. PA, physical activity; MVPA, moderate-to-vigorous physical activity; ST, screen time. Meeting physical activity guidelines means accumulating 180 min of total PA per day of which 60 minutes are MVPA. Meeting screen time guidelines means accumulating up to 1 hour of screen time per day. *p*-values were derived from generalised linear models with city and sex (and, when appropriate, their interaction and accelerometer wear time) as predictors of the variables listed in the table.
