# Supplementary material for: Parent-perceived neighbourhood environment, parenting practices and preschool-aged children physical activity and screen time: a cross-sectional study of two culturally and geographically diverse cities
Source: BMC Pediatr. 2022 May 27;22:309. doi: 10.1186/s12887-022-03377-0 (PMC9137173; doi:10.1186/s12887-022-03377-0)
Supplement: Supplementary file 3 — Additional file 3: Table S3. Interaction effects between parent-perceived neighbourhood physical activity places and facilities, safety-related attributes and physical-activity-related parenting practices on preschool-aged children’s physical activity and screen time. [file 12887_2022_3377_MOESM3_ESM.docx]

| **Neighbourhood environmental attribute** | **Moderator** | **Measurement period** | **Total PA**  [accelerometer-assessed] | **MVPA**  [accelerometer-assessed] | **Screen time**  [parent-reported] |
| --- | --- | --- | --- | --- | --- |
|  |  |  | *b* (95% CI) | e*^b^* (95% CI) | *b* (95% CI) |
| Availability of places for children’s PA | Traffic hazards | Weekday | -1.41 (-4.85, 2.02) | 1.006 (0.822, 1.229) | -0.05 (-0.23, 0.13) |
|  |  | Weekend | -0.52 (-4.28, 3.24) | 0.998 (0.972, 1.025) | 0.02 (-0.21, 0.25) |
|  | Signs of physical and social disorder | Weekday | 0.71 (-1.87, 3.28) | 1.013 (0.989, 1.017) | 0.29 (-0.22, 0.80) |
|  |  | Weekend | 1.71 (-1.68, 5.10) | 1.009 (0.985, 1.032) | 0.14 (-0.32, 0.59) |
|  | Community cohesion | Weekday | -2.12 (-5.25, 1.00) | 0.993 (0.969, 1.019) | -0.06 (-0.21, 0.08) |
|  |  | Weekend | -2.48 (-5.68, 0.73) | 0.988 (0.982, 1.095) | -0.15 (-0.12, 0.25) |
|  | Informal social control – education and supervision of children | Weekday | 0.09 (-2.66, 2.85) | 1.003 (0.980, 1.028) | -0.04 (-0.17, 0.10) |
|  |  | Weekend | 0.51 (-2.53, 3.55) | 0.998 (0.974, 1.022) | -0.02 (-0.16, 0.13) |
|  | Informal social control – civic engagement for neighbourhood enhancement | Weekday | 1.54 (-1.86, 4.94) | **1.029 (1.004, 1.055)^*^** | -0.07 (-0.24, 0.10) |
|  |  | Weekend | **4.08 (0.49, 7.68)^*^** | 1.028 (0.998, 1.058) | -0.13 (-0.27, 0.01) |
|  |  |  |  |  |  |
| Availability of active-play equipment | Traffic hazards | Weekday | -2.74 (-7.48, 2.00) | 1.007 (0.972, 1.043) | -0.03 (-0.27, 0.21) |
|  |  | Weekend | **-6.56 (-12.41, -0.71)^*^** | 0.976 (0.934, 1.019) | -0.05 (-0.34, 0.25) |
|  | Signs of physical and social disorder | Weekday | 1.53 (-1.75,4.81) | 1.015 (0.982, 1.048) | -0.03 (-0.21, 0.15) |
|  |  | Weekend | 1.74 (-2.01, 5.50) | 1.020 (0.987, 1.053) | -0.15 (-0.39, 0.09) |
|  | Community cohesion | Weekday | -0.26 (-3.26, 2.74) | 1.013 (0.985, 1.041) | -0.02 (-0.20, 0.15) |
|  |  | Weekend | 0.55 (-2.83, 3.94) | 1.001 (0.975, 1.027) | 0.14 (-0.08, 0.36) |
|  | Informal social control – education and supervision of children | Weekday | 0.70 (-3.22, 4.63) | 1.011 (0.984, 1.070) | 0.03 (-0.18, 0.23) |
|  |  | Weekend | 0.17 (-3.42, 3.76) | 0.995 (0.967, 1.024) | 0.09 (-0.13, 0.31) |
|  | Informal social control – civic engagement for neighbourhood enhancement | Weekday | 0.57 (-3.33, 4.46) | 1.013 (0.987, 1.040) | 0.06 (-0.15, 0.26) |
|  |  | Weekend | 0.55 (-3.71, 4.82) | 0.993 (0.963, 1.024) | -0.03 (-0.26, 0.19) |
|  |  |  |  |  |  |
| Availability of places for children’s PA | Parental engagement | Weekday | **-3.77 (-6.66, -0.87)^**^** | 0.991 (0.967, 1.015) | -0.04 (-0.20, 0.13) |
|  |  | Weekend | -1.19 (-5.41, 3.02) | 0.993 (0.963, 1.024) | -0.04 (-0.22, 0.14) |
|  |  |  |  |  |  |
| Availability of active-play equipment | Parental engagement | Weekday | 3.79 (-0.17, 7.75) | **1.036 (1.006, 1.067)^*^** | -0.12 (-0.31, 0.07) |
|  |  | Weekend | 0.47 (-4.23, 5.15) | 1.019 (0.987, 1.051) | -0.20 (-0.44, 0.04) |
|  |  |  |  |  |  |
| Traffic hazards | Restrictions for safety concerns | Weekday | -4.98 (-12.81, 2.84) | 0.964 (0.910, 1.022) | 0.20 (-0.13, 0.53) |
|  |  | Weekend | 0.20 (-8.44, 8.84) | 0.967 (0.905, 1.033) | 0.29 (-0.12, 0.69) |
|  |  |  |  |  |  |
| Signs of physical and social disorder | Restrictions for safety concerns | Weekday | 0.50 (-6.38, 7.37) | 1.013 (0.958, 1.071) | 0.06 (-0.27, 0.40) |
|  |  | Weekend | 4.98 (-3.47, 13.42) | 1.019 (0.958, 1.083) | **0.54 (0.16, 0.92)^**^** |

*Notes.* *b*, estimate of unstandardised regression coefficient; e*^b^*, exponentiated estimate of unstandardised regression coefficient; CI, confidence interval; PA, physical activity; MVPA, moderate-to-vigorous physical activity. ^*^ *p*<.05. All models were adjusted for socio-demographic characteristics. Models of total PA and MVPA were adjusted for average accelerometer wear time. Regression coefficients of neighbourhood environmental attributes moderated by other environmental attributes were not adjusted for PA-related parenting practices. ^*^ *p*<.05; ^**^ *p*<.01.
